# Emergence of Carbapenemase Producing *Klebsiella Pneumonia* and Spread of KPC-2 and KPC-17 in Taiwan: A Nationwide Study from 2011 to 2013

**DOI:** 10.1371/journal.pone.0138471

**Published:** 2015-09-18

**Authors:** I-Ling Tseng, Yu-Mei Liu, Shiow-Jen Wang, Hung-Yi Yeh, Chia-Lun Hsieh, Hsueh-Lin Lu, Yu-Chuan Tseng, Jung-Jung Mu

**Affiliations:** 1 Bacterial Enteric and Emerging Diseases Laboratory, Center for Research, Diagnostics and Vaccine Development, Centers for Disease Control, Taipei, Taiwan; 2 Bioengineering Group, Development Center for Biotechnology, Taipei, Taiwan; Cornell University, UNITED STATES

## Abstract

The emergence of carbapenemase-producing *Klebsiella pneumoniae* (CPKP) has become a great concern worldwide. In this study, 994 non-duplicate, carbapenem non-susceptible *Klebsiella pneumonia* isolates were collected in Taiwan from 2011 to 2013 for detection of the carbapenemase genes, assessment of antimicrobial susceptibility and molecular epidemiology studies. Of these 994 isolates, 183 (18.4%) had carbapenemase genes: 157 (15.8%) KPC (145 KPC-2 and 12 KPC-17), 16 (1.6%) IMP-8, 9 (0.9%) VIM-1, and 1 (0.1%) NDM-1. KPC had the highest prevalence rate among the carbapenemases and represented a major epidemic clone circulating in Taiwan. The ST512 and ST258 KPC-2 KPs were first identified in Taiwan and were grouped into a small cluster in the PFGE profile. In addition, the genetic structure encompassing the *bla*_KPC_ gene of the ST512 and ST258 isolates showed a different pattern from that of other KPC isolates. ST11 may be a major sequence type circulating in Taiwan, although a specific minor clone has begun to be observed. This is the first report of ST258 and ST512 KPC-2 KP isolates in Taiwan, whether ST258 and ST512 will become the next endemic problems in Taiwan should be closely monitored.

## Introduction

The emergence and global spread of carbapenemase-producing *Enterobacteriaeae* (CPE) is of great concern to health services worldwide [1–3]. *Klebsiella pneumonia* (KP), which commonly acquires resistance encoded on mobile genetic elements, including ones that encode carbapenemases, is a prime example. Recently, carbapenemase- producing *Klebsiella pneumoniae* (CPKP) has rapidly emerged as one of the major nosocomial pathogens among CPE [4, 5].

Carbapenemases, which readily hydrolyze carbapenems, have been divided into four classes of beta-lactamases, with the epidemiologically relevant carbapenemases falling into three of these [6]: class A beta-lactamases (NMC, IMI, SME, KPC, GES), class B, metallo-beta-lactamases (IMP, VIM, GIM, SPM, NDM) and class D beta-lactamases (OXA) [7]. These carbapenemases have emerged in various parts of the world, including Europe, the US, the Indian subcontinent, and eastern countries. *Klebsiella pneumoniae* with KPC carbapenemase was first detected in 1996 in the US and subsequently spread worldwide [8–10]. NDM-1 was first described in *Klebsiella pneumoniae* in 2009 and has since attained an international distribution [11, 12]. The high prevalence of CPKP makes it necessary to investigate resistance epidemiology in each country to combat further spread.

Although reports of CPKP isolates have been increasing dramatically in Taiwan, with the highest prevalence among CPE [13–16], the recent longitudinal nationwide data have not been thoroughly investigated. The purpose of this study was to delineate the resistance profiles, carbapenemase genes and molecular epidemiology of CPKP isolates obtained from 2011 to 2013, as these data may help us to understand the current status of CPKP spread and to assist in determining infection control measures.

## Material and Methods

### Bacterial isolates

Centers for Disease Control, Taiwan (Taiwan CDC) encouraged hospitals to submit their carbapenem non-susceptible *Enterobacteriaceae* isolates for molecular and epidemiological characterization. From January 2011 to December 2013, a total of 994 non-duplicate, carbapenem non-susceptible *Klebsiella pneumonia* isolates (MIC > 1 mg/L for imipenem or meropenem or MIC > 0.5 mg/L for ertapenem) were reported from 28 hospitals in northern (n = 11), central (n = 7) and southern (n = 10) Taiwan. The isolates were identified by standard microbiological methods using the BD Phoenix™ Automated Microbiology System. This study was conducted in accordance with the Declaration of Helsinki and was approved by the Ethics Committee of the Taiwan CDC (Project No. 102016 and 104126). The informed consent procedure was exempted by law when doctors reported cases through the National Communicable Disease Surveillance Systems to the Taiwan CDC.

### Antimicrobial susceptibility testing

Antimicrobial susceptibility of all of the isolates were determined by broth microdilution (BD Phoenix™ Automated Microbiology System with phoenix NMIC/ID-72 panel, Becton Dickinson Diagnostic Systems, Sparks, MD, USA) and interpreted according to the CLSI guidelines released in Jan. 2012 (M100-S22). For tigecyclin, the MIC breakpoint used for susceptibility was determined by E-test based on the criteria proposed by the U.S. Food and Drug Administration (FDA) (susceptible, MICs ≤ 2 mg/L). For colistin, the MIC breakpoint used for susceptibility was defined by the European Committee on Antimicrobial Susceptibility Testing (EUCAST-2012) criteria (susceptible: MICs ≤ 2 mg/L).

### Determination of carbapenemase genes

Genes encoding different classes of carbapenemases, including class A (KPC, NMC, SME, IMI, and GES), class B (NDM, IMP, VIM, SPM, GIM, and SIM), and class D (OXA-48), were PCR identified as previously described [7, 17].

### Plasmid analysis

Plasmid DNA was extracted from KPC-2 and KPC-17 KPs with the Qiagen Midi Kit (Qiagen). *HindIII* digested plasmids were separated in 0.8% agarose and transferred to nylon membranes for Southern hybridization. KPC-containing fragments were identified by hybridization with Dig-labeled *bla*_KPC_-specific probe generated by the DIG High Prime DNA Labeling and Detection Starter Kit II (Roche Applied Sciences, Germany). The images were visualized by using the VersaDoc Imaging System 4000MP (Bio-Rad, USA).

The extracted KPC-2- and KPC-17-producing plasmids described above were also transformed into NEB 10-beta electrocompetent *E*.*coli* recipients (C3020K, BioLabs) by using the Gene Pulser Xcell™ electroporation system (Bio-Rad). Transformants were confirmed to have *bla*_kpc-2_ or *bla*_KPC-17_ by PCR and sequencing analysis. Extracted plasmid DNA from the transformants was digested with *HindIII* and separated in a 0.8% agarose gel. The agarose gel was stained with ethidium bromide and visualized by using the Gel Doc™ XR+ system (Bio-Rad, USA).

### Molecular typing

The genotypes of isolates harboring carbapenemase genes were determined by using pulsed-field gel electrophoresis (PFGE) analysis. The genetic relatedness of the test strains was generated by the unweighted pair-group method (UPGMA). Isolates that exhibited a PFGE profile with more than 80% similarity (pulsotype, PT) were considered as closely related strains. MLST was performed on *K*. *pneumoniae* isolates and seven housekeeping genes (i.e., *rpoB*, *gapA*, *mdh*, *pgi*, *phoE*, *infB* and *tonB*) were amplified. The resultant PCR products were purified and sequenced. Sequence types (STs) were assigned using online database tools according to the Institut Pasteur MLST databases (http://www.pasteur.fr/recherche/genopole/PF8/mlst/Kpneumoniae.html).

## Results

### Characteristics of carbapenemase-producing KP

Between 2011 and 2013, a total of 994 non-duplicate, carbapenem non-susceptible *Klebsiella pneumonia* isolates were reported to the Taiwan CDC. The presence of genes encoding carbapenemases was confirmed by PCR and DNA sequencing. Of these 994 isolates, 183 (18.4%) had carbapenemase genes detected from the following groups: 157 (15.8%) from KPC (145 from KPC-2 and 12 from KPC-17), 16 (1.6%) from IMP-8, 9 (0.9%) from VIM-1, and 1 (0.1%) from NDM-1 (Table 1). Although IMP-8 KP had been reported a decade before [18], its frequency has been largely surpassed recently by the rapid emergence of KPC-KP.

**Table 1:** No. of carbapenem non-susceptible KP isolates.

Carbapenemase Group (n^a^)	Carbapenemase variants	2011	2012	2013	total	total
**KPC (157)**	**KPC-2**	**26**	**72**	**47**	**157 (15.8%)**	**145 (14.6%)**
	**KPC-17**	**0**	**8**	**4**		**12 (1.2%)**
**IMP (16)**	**IMP-8**	**5**	**4**	**7**	**16 (1.6%)**	
**VIM (9)**	**VIM-1**	**0**	**3**	**6**	**9 (0.9%)**	
**NDM (1)**	**NDM-1**	**0**	**0**	**1**	**1 (0.1%)**	
**total CPKP (183)**		**31 (9.5%)**	**87 (25.4%)**	**65 (20%)**	**183 (18.4%)**	
**total carbapenem non-susceptible KPs (994)**		**326**	**343**	**325**	**994**	

^a^n: isolates numbers in each carbapenemase group

Of the 183 cases that had carbapenemase-producing KP isolates, the clinical features from 163 cases showed that the isolates were primarily recovered from urine (36.2%), followed by sputum (20.9%), blood (14.1%), and wound and rectal swabs (7.4%). (Table 2). Ninety percent of the patients were hospitalized with underlying diseases, and 65% had received antibiotic treatment within the last 6 months (Table 2).

**Table 2:** Clinical features of patients with carbapenemase producing KP isolates from clinical culture.

Carbapenemase Types		KPC-2	KPC-17	IMP	VIM	NDM	Total Number
**Culture Site**	**blood**	**17**	**3**	**2**	**1**	**0**	**23 (14.1%)**
	**urine**	**46**	**5**	**4**	**4**	**0**	**59 (36.2%)**
	**sputum**	**26**	**2**	**5**	**1**	**0**	**34 (20.9%)**
	**wound**	**10**	**0**	**0**	**1**	**1**	**12 (7.4%)**
	**rectal swab**	**11**	**0**	**0**	**1**	**0**	**12 (7.4%)**
	**body fluid**	**3**	**1**	**0**	**0**	**0**	**4 (2.5%)**
	**CVP tip**	**7**	**1**	**0**	**0**	**0**	**8 (5%)**
	**multiple sites**	**10**	**0**	**0**	**0**	**0**	**10 (6.1%)**
	**Ear**	**0**	**0**	**0**	**1**	**0**	**1 (0.61%)**
**Hospitalization**		**119**	**10**	**10**	**7**	**1**	**147 (90.2%)**
**Underlying Diseases** ^**a**^		**117**	**11**	**11**	**8**	**1**	**148 (90.8%)**
**Previous Antibiotic Treatment** ^**b**^		**85**	**5**	**10**	**5**	**1**	**106 (65%)**
**Travel Outside Taiwan**		**1** ^c^	**0**	**0**	**0**	**0**	**1 (0.61%)**	

^a^heart disease, stroke, cancer, chronic respiratory diseases, chronic renal dysfunction, diabetes, hypertension, liver cirrhosis, epatitis

^b^Received antibiotics treatment within 6 months

^c^Visited country: China

### Susceptibility testing

Susceptibility to antibiotics is depicted in Table 3, which shows that all CPKPs were non-susceptible to ertapenem, ampicillin, cephazolin, and cefepime. Among these, the KPC-KPs and one NDM-KP were non-susceptible to all three carbapenem antibiotics, but of the IMP-KPs and VIM-KPs, 81.3% and 22.2% were susceptible to meropenem, respectively. Comparing the susceptibility rates with fluoroquinolones, KPC-KPs were non-susceptible to both ciprofloxacin and levofloxacin, and NDM-KP was non-susceptible only to ciprofloxacin. In contrast, IMP-KPs and VIM-KPs showed susceptibility rates to these two fluoroquinolone antibiotics from 22.2% to 100%.

**Table 3:** The susceptibility of CPKPs.

Antibiotics	KPC-2	KPC-17	IMP-8	VIM-1	NDM-1
	Susceptibility (%)	Susceptibility (%)	Susceptibility (%)	Susceptibility (%)	Susceptibility (%)
**ERT**	0	0	0	0	0
**IMP**	0	0	43.8	0	0
**MER**	0	0	81.3	22.2	0
**AMK**	69.0	100	68.8	100	100
**GEN**	60.0	50.0	43.8	33.3	100
**AZT**	0	0	43.8	0	0
**SXT**	59.3	50.0	25.0	0	0
**AMP**	0	0	0	0	0
**CFZ**	0	0	0	0	0
**CFP**	0	0	0	0	0
**CPX**	0	0	25.0	22.2	0
**LEV**	0	0	37.5	100	100
**COL**	98.6	91.7	100	100	100
**TGC**	95.9	100	43.8	100	100

Susceptibility rates of all of the CPKP isolates to aminoglycoside antibiotics (amikacin and gentamycin) ranged from 33% to 100%. In addition, all CPKPs isolates revealed high-level tigecycline and colistin susceptibility (more than 90%), except that IMP-KPs had a lower susceptibility rate (43.8%) to tigecycline.

### Phylogenetic analysis

**Fig 1:**
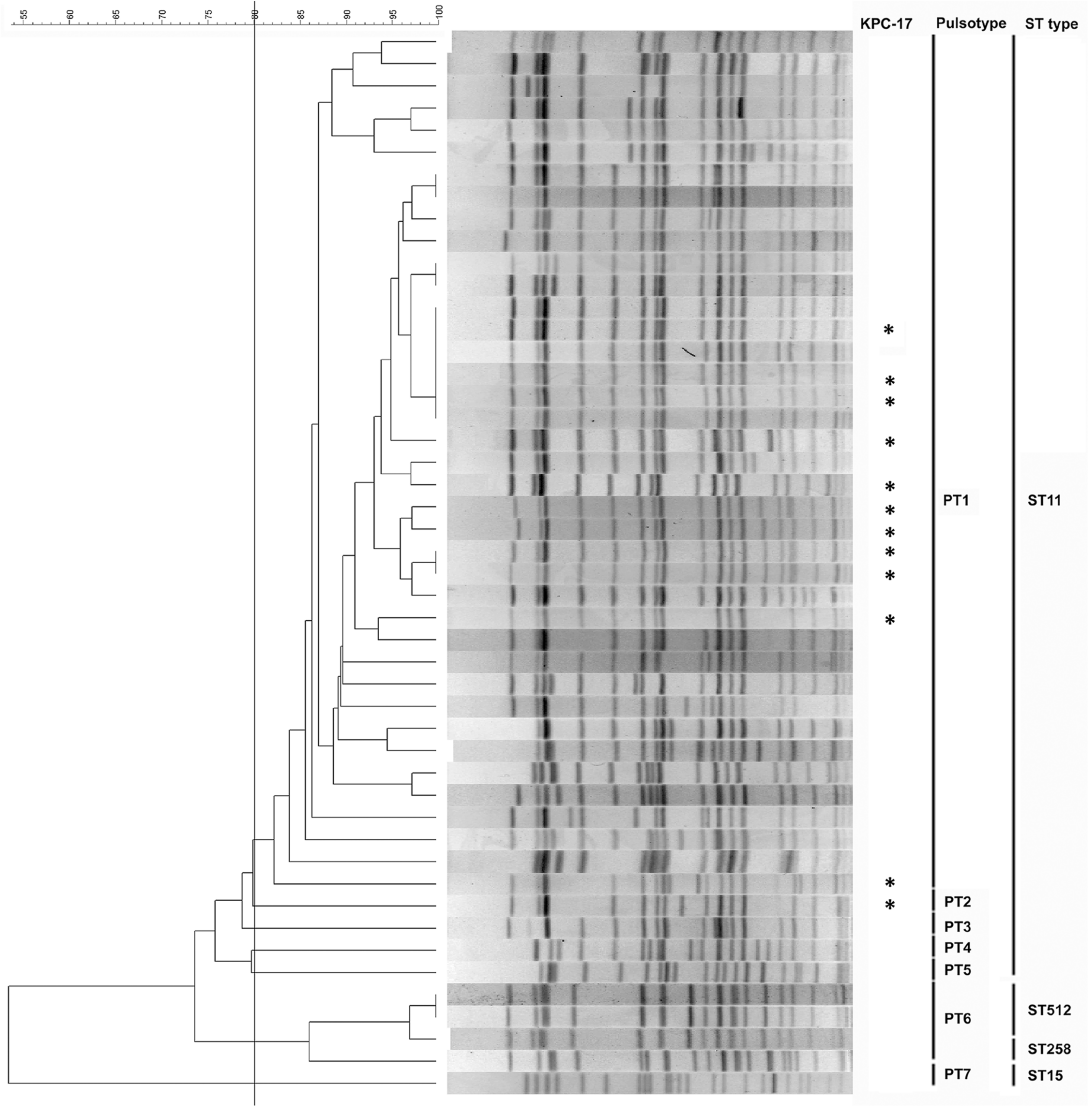
The relatedness of KPC-KPs. PFGE dendrogram with the corresponding MLST sequence types of 48 KPC-KP isolates. Isolates that exhibited PFGE profiles with more than 80% similarity were considered as one pulsotype, PT. Asterisk represented KPC-17 KP.

PFGE of 157 KPC-KP isolates identified 7 pulsotypes, pulsotype (PT) PT1 to PT7 using 80% similarity as the cut-off (S1 Fig). PT1, the major cluster, included 137 KPC-2 KPs and 11 KPC-17 KPs, PT2 included one KPC-17 isolate, and PT3-7 included 8 KPC-2 KP isolates. Fig 1 showed the PFGE profile with the corresponding MLST sequence types of 48 KPC-KP isolates, including 28 KPC-2 KPs (belonging to PT1, one isolate from each hospital), 12 KPC-17 KPs (11 belonging to PT1, one belonging to PT2) and 8 KPC-2 KPs belonged to PT3-7 isolates (Fig 1). The MLST sequence types of the PT1-5 isolates in Fig 1 were all ST11, including 12 KPC-17 isolates. PT6, the second major cluster, included 4 KPC-2 KPs. Among the PT6 isolates, three collected from the same hospital were sequence type ST512 and the fourth collected from another hospital was ST258. Both hospitals were localized in southern part of Taiwan. The KPC-2 isolate belonging to PT7 was sequence type ST15 and the patient having this isolate had travel history outside the country (Table 3).

**Fig 2:**
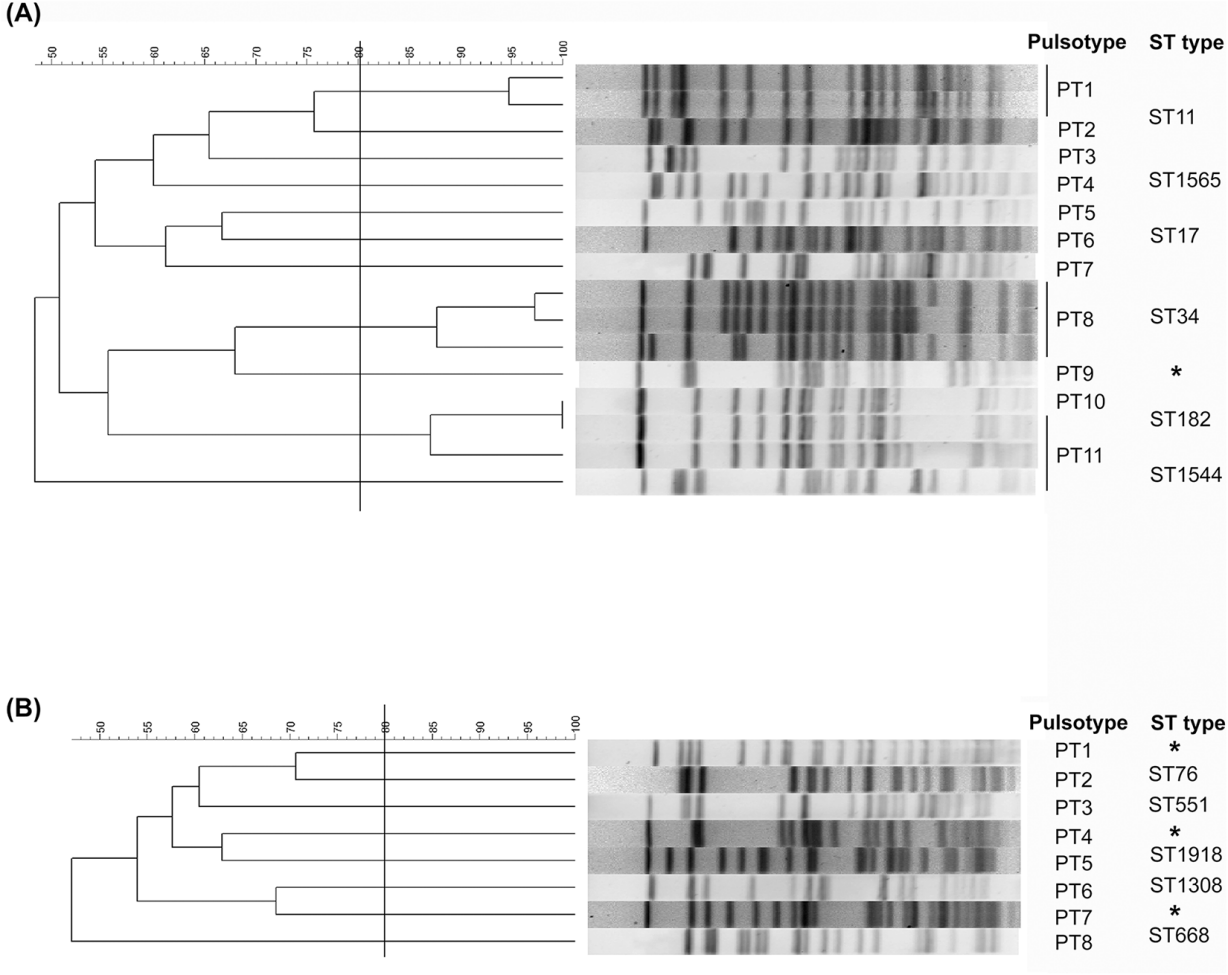
The relatedness of (A) IMP and (B) VIM KPs. PFGE dendrogram with the corresponding MLST sequence types of **(A)** 16 IMP-KP and **(B)** 8 VIM-KP isolates. Isolates that exhibited PFGE profiles with more than 80% similarity were considered as one pulsotype. Asterisk represented novel ST types of IMP-KP and VIM-KP that have been submitted and are awaiting confirmation from curator.

PFGE grouped 16 isolates of IMP-8 KPs into 11 pulsotypes (designated pulsotypes PT1 to PT11) using the 80% similarity as the cut-off (Fig 2A). Three clusters were identified in PT1, PT8 and PT10 and each pulsotype in the cluster corresponded to a specific MLST sequence type. The three clusters obtained from 3 different hospitals were located in central, northern and southern Taiwan, respectively. For VIM-1 KPs, PFGE identified 8 pulsotypes, with each pulsotype containing a single isolate (Fig 2B). A novel ST type corresponding to PT9 from the IMP-KP and three novel ST types from the VIM-KP isolates have been submitted to the Institut Pasteur MLST database and are awaiting confirmation from the curator to assign sequence type.

### Genetic analysis of KPC producing plasmids

**Fig 3:**
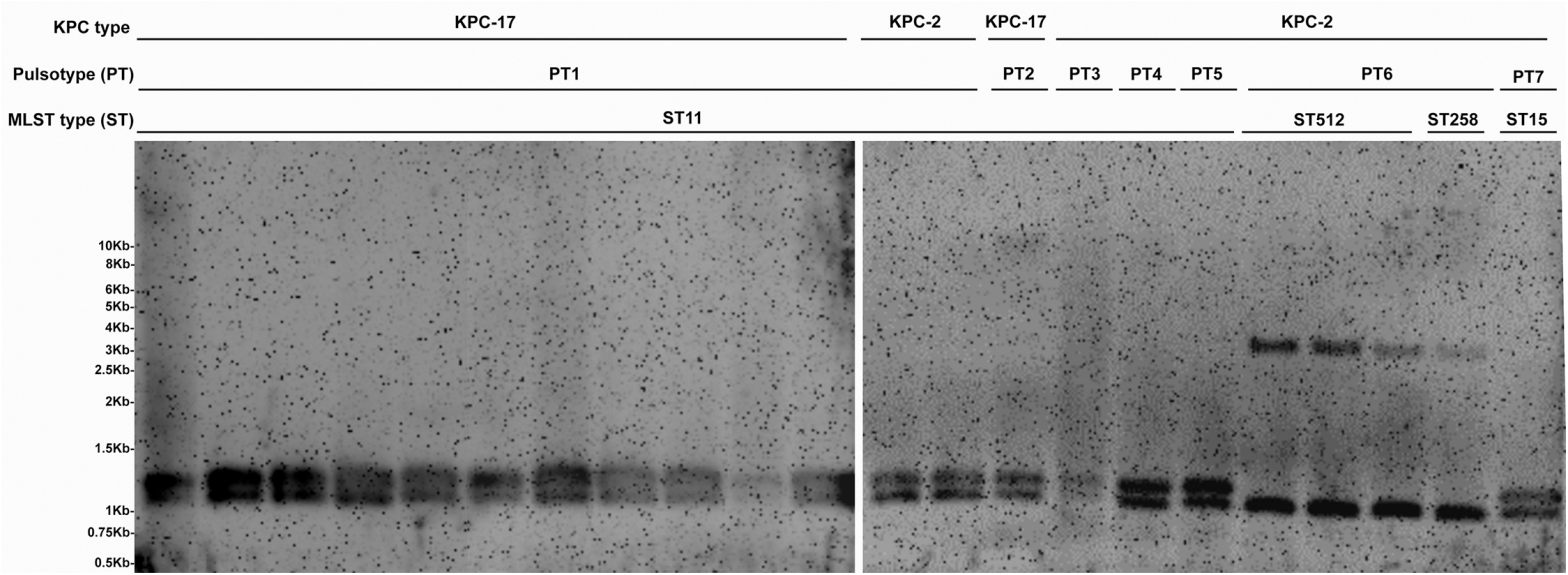
Southern hybridization depicting the size patterns encompassing the *bla*_KPC_ gene. HindIII digested plasmids from each pulsotype of KPC-2 and all of the KPC-17 isolates were detected by Southern hybridization with a Dig-labeled *bla*_KPC-2_ gene as probe.

To analyze the genetic structure encompassing the *bla*_KPC-2_ gene, plasmid extraction, *HindIII*-restriction mapping and Southern hybridization with the *bla*_KPC-2_ gene were carried out on each pulsotype of KPC-2 and one all of the KPC-17 isolates (Fig 3). The result revealed that isolates from PT1-5 and PT7, including all KPC-17 isolates, had similar size patterns that encompassed the *bla*_KPC_ gene. However, isolates from PT6 with ST512 and ST258 MLST types showed different patterns from those of other PTs.

**Fig 4:**
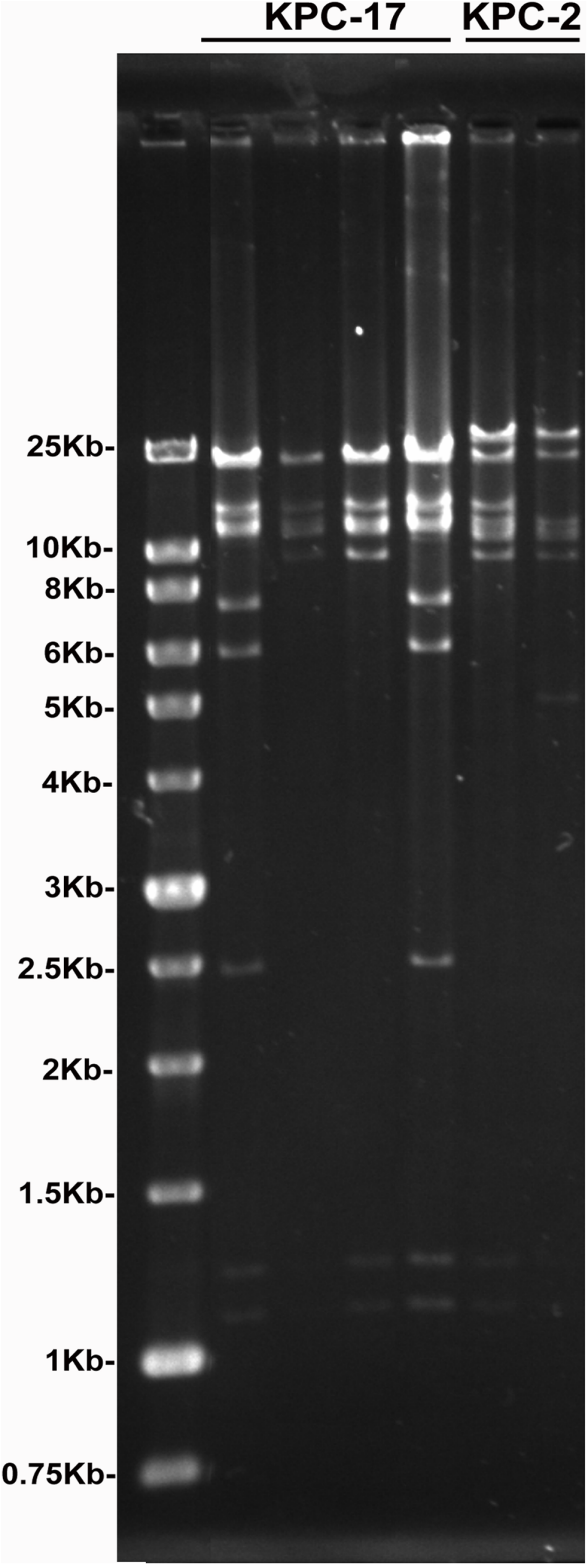
Plasmid DNA profiles from 4 KPC-17 and 2 KPC-2 isolates in PT1. *HindIII* restriction mapping of transformed plasmids from 4 KPC-17 and 2 KPC-2 isolates selected from PT1.

Transformation of KPC producing plasmids from 4 KPC-17 and 2 KPC-2 isolates selected from PT1 was used for mapping the genetic patterns of KPC producing plasmids. *HindIII* restriction map from the transformed plasmids showed different plasmid DNA profiles among the selected KPC-17 and KPC-2 isolates (Fig 4), suggesting multiple types of KPC-2- and KPC-17-producing plasmids spread in Taiwan.

## Discussion

The first reported CPKP outbreak detected in Taiwan was caused by IMP-8 KPs through 1999 to 2000 [18]. There were no KPC, NDM and OXA carbapenemases detected in *K*. *pneumonia* before 2010 [19]. The first KPC-2 KPs outbreak in Taiwan was not reported until 2011, and the first NDM-1 KP was also identified in the same year [20, 21]. Since then, the CPKPs have dramatically increased from 9.5% to 25.4% in 2011 and 2012 (Table 1), and the prevalence rate of IMP-8 has been greatly surpassed by the rapid emergence of KPC-KPs (1.6% *v*.*s*. 15.8%) (Table 1). The DNA restriction profiles of the KPC-producing plasmids revealed diverse genetic backbones among the KPC-KP isolates (Fig 4), suggesting that different plasmids harboring the *bla*_KPC_ gene have already become widespread among KP strains in Taiwan.

ST11 KPC-2 KPs has been the predominant clone since the first outbreak occurred in 2011 in Taiwan, which is also support by our surveillance results [14, 20]. In this study, 28 KPC-2 isolates, selected from each hospital and 12 KPC-17 isolates were identified as sequence type ST11 (Fig 1). However, a minor cluster in the PFGE profile, PT6, including four isolates with sequencing type ST512 and ST258, were detected (Fig 1). ST258 is a major KPC-producing clone throughout the world [22–24] and ST512 is the predominant clone primarily carrying the *bla*_KPC-3_ gene [25, 26]. This is the first report of ST512 and ST258 KPC-2 KPs identified in Taiwan, and the four cases with ST258 and ST512 isolates did not have a history of travel outside of the country. The analysis of the genetic structure encompassing the *bla*_KPC-2_ gene showed different size patterns between ST258, ST512 and other KPC-KP isolates (Fig 3). As the ST258 and ST512 isolates have been disseminated worldwide, whether they will be the next endemic problems in Taiwan should to be closely monitored.

OXA-producing KP was not detected in our nationwide investigation or by other national and regional surveillance [14, 19, 27], but was recently report detected in one hospital in Taiwan [13]. Although the *bla*_OXA_ type was not clearly identified in the associated report and seemed to thus far be localized to only one hospital, the possible dissemination and emerging problems of *bla*_OXA_ in the future should be monitored.

Our results are based on a large representative sample of CPE cases in Taiwan, however, reporting of CPE is not mandatory in this country so far. It is remarkable that, from 2011 to 2013, the number of KPC-KP isolate increased dramatically. This suggest that a recent epidemiological change may have occurred in Taiwan, characterized by a clonal spread of a specific ST11 sequence type of KPC-KP. In addition, ST11 sequence type was also Fdetected as a major MLST clone in IMP-8 KPs in previous and this study [19], indication ST11 sequencing type may play an important role in dissemination of MBL genes in Taiwan.

## Supporting Information

S1 Fig The profile of KPC-KPs. PFGE profile of 157 KPC-KP isolates. Isolates that exhibited PFGE profiles with more than 80% similarity were considered as one pulsotype, PT.
(TIF)
