# Supplementary figures and images for: Emergence of Carbapenemase Producing Klebsiella Pneumonia and Spread of KPC-2 and KPC-17 in Taiwan: A Nationwide Study from 2011 to 2013
Source: PLoS One. 2015 Sep 18;10(9):e0138471. doi: 10.1371/journal.pone.0138471 (PMC4575059; doi:10.1371/journal.pone.0138471)

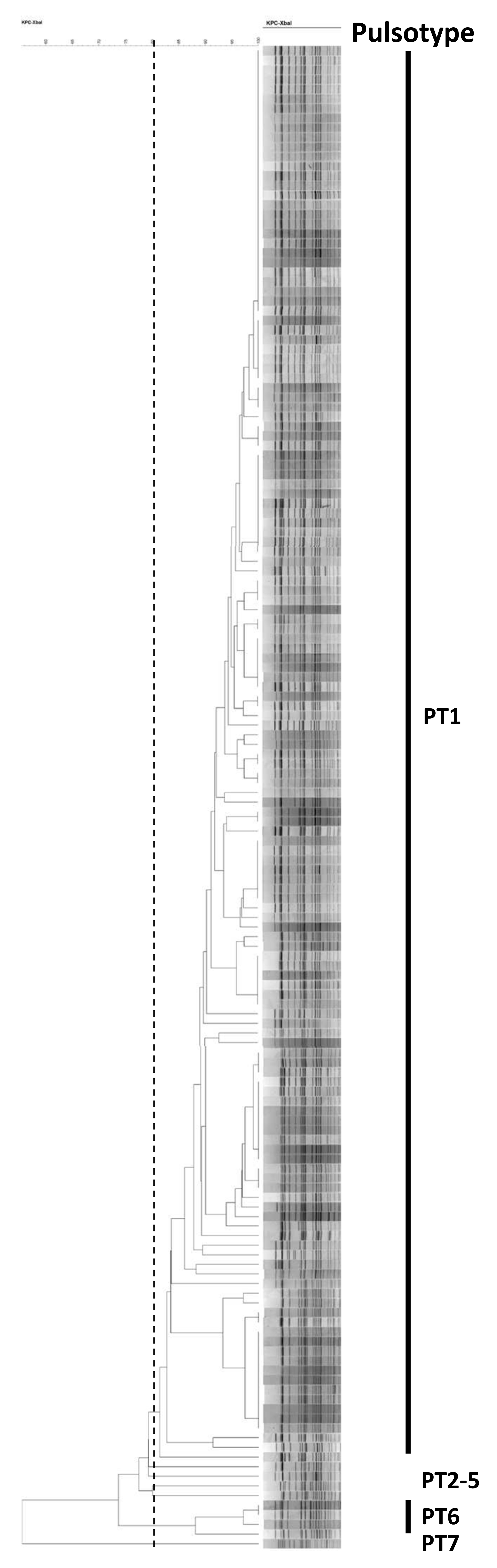

Supplement: S1 Fig — PFGE profile of 157 KPC-KP isolates. Isolates that exhibited PFGE profiles with more than 80% similarity were considered as one pulsotype, PT. (TIF) [file pone.0138471.s001.TIF]
